# Study of the variations of fall induced hip fracture risk between right and left femurs using CT-based FEA

**DOI:** 10.1186/s12938-017-0407-y

**Published:** 2017-10-03

**Authors:** Tanvir R. Faisal, Yunhua Luo

**Affiliations:** 1 Department of Physical Medicine and Rehabilitation, Feinberg School of Medicine, Northwestern University, Chicago, IL 60611 USA; 2 Legs + Walking Lab, Shirley Ryan AbilityLab, Chicago, IL 60610 USA; 3 Department of Mechanical Engineering, Faculty of Engineering, University of Manitoba, Winnipeg, MB R3T 2N2 Canada

**Keywords:** Quantitative Computed Tomography, Fracture risk indicator, Hip fracture, Single-stance, Sideways fall

## Abstract

**Background:**

Hip fracture of elderly people—suffering from osteoporosis—is a severe public health concern, which can be reduced by providing a prior assessment of hip fracture risk. Image-based finite element analysis (FEA) has been considered an effective computational tool to assess the hip fracture risk. Considering the femoral neck region is the weakest, fracture risk indicators (FRI) are evaluated for both single-legged stance and sideways fall configurations and are compared between left and right femurs of each subject. Quantitative Computed Tomography (QCT) scan datasets of thirty anonymous patients’ left and right femora have been considered for the FE models, which have been simulated with an equal magnitude of load applied to the aforementioned configurations. The requirement of bilateral hip assessment in predicting the fracture risk has been explored in this study.

**Results:**

Comparing the sideways fall and single-legged stance, the FRI varies by 64 to 74% at the superior aspects and by 14 to 19% at the inferior surfaces of both the femora. The results of this in vivo analysis clearly substantiate that the fracture is expected to initiate at the superior surface of femoral neck region if a patient falls from his/her standing height. The distributions of FRI between the femurs vary considerably, and the variability is significant at the superior aspects. The *p* value (= 0.02) obtained from *paired sample t*-*Test* yields p value ≤ 0.05, which shows the evidence of variability of the FRI distribution between left and right femurs. Moreover, the comparison of FRIs between the left and right femur of men and women shows that women are more susceptible to hip fracture than men.

**Conclusions:**

The results and statistical variation clearly signify a need for bilateral hip scanning in predicting hip fracture risk, which is clinically conducted, at present, based on one hip chosen randomly and may lead to inaccurate fracture prediction. This study, although preliminary, may play a crucial role in assessing the hip fractures of the geriatric population and thereby, reducing the cost of treatment by taking predictive measure.

## Background

One of the fatal injuries observed in elderly people is hip fracture, which is the most serious complication of osteoporosis. Due to reduction in bone mass with age, a sideways fall from the standing height often triggers the hip fracture associated with a high level of morbidity and mortality in elderly population. Hence, the elder people often undergo screening to identify the quality of bone by densitometric technique, such as Dual-energy X-ray absorptiometry (DXA) [1], which is recognized by the World Health Organization (WHO). During a clinical densitometric screening, only one hip is typically scanned instead of bilateral hips for osteoporosis assessment, reporting a good correlation between the bone mineral density (BMD) of left and right hips. However, an accurate assessment of fracture risk is a vital step for initial screening, which can help reducing fall induced hip fracture by providing and creating a prior awareness to the patients.

Although DXA is primarily used as the reference method to measure BMD, the BMD based fracture assessment lacks desired accuracy. For instance, half of the patients who suffer from fracture have BMD above the conventional osteoporotic threshold [2]. Accepting the fact, the DXA is still used clinically concerning lower potential health risk due to radiation dose and associated cost [1, 3], and only one hip is generally scanned for the assessment. It is argued that scanning of bilateral hips requires additional time to repositioning a patient and thereby, exposing the individual to unnecessary radiation. Compared with the early/first generation scanning systems, the modern scanning modalities are fast; and a single hip can be scanned in a few minutes. The scanning of the second hip in an automated sequential bilateral hip mode adds only about 1–2 min, and both hips can be scanned in less than 5 min after the patient is positioned correctly [4–8]. Thus, the ability to perform bilateral hip scans may not be constrained by time issues. Albeit, clinical practice is to scan patient’s “non-dominant hip” (determined by asking the patient if he or she is right- or left- handed) irrespective of imaging modalities to assess the hip fracture risk.

Several studies have been conducted on subjects with different race [9], sex [10], age [11] to justify the requirement of scanning one or both femurs for assessing osteoporosis to predict hip fracture [12, 13]. A number of studies have shown a significant correlation between the two hip BMD measurements at femoral neck of normal Chinese women [6], and the Caucasian women of age above 65 years [8]. On the contrary, no significant difference has also been reported for the groups of 36 healthy women of the United Kingdom and the USA [14, 15]. Although these studies show no systematic differences between the hips, the correlation may not be held true for a larger population of patients. However, researchers have also observed BMD variation between the femora [14, 16, 17], where the variation may be originated due to unilateral hip disorders, arthritis, hemiplegia and osteoporosis. It is still unclear whether osteoporotic hip fracture can be assessed equally or not, conducting bilateral scanning of both left and right femora. Nevertheless, the decision of scanning mostly depends on technologist’s personal preference and the physical location of the scanner, even though single leg scanning may underestimate the osteoporosis and thereby the fracture [17].

Until today, to the best of our knowledge, only densitometric studies have been conducted to investigate the necessity for mono or bilateral hip scanning. Due to the nature of 2D imaging modality, bone mass measured by DXA fail to incorporate the essential factors such as geometry, microarchitecture, and material properties of bone tissue [18] as well as loading condition as a consequence of falling. Hence, it is important to investigate the requirement of mono or bilateral hip assessment in predicting the risk of hip fracture considering more robust imaging technique—Quantitative Computed Tomography (QCT). The QCT can predict the strength of femoral bone more accurately since the imaging modality can adequately capture the bone’s 3D anatomic structure. The strength of a femoral bone largely depends on its 3D anatomic structure, which is not correctly reflected in DXA-based FEA. The strength of the femoral bone predominantly depends on its geometry and structural property, the distribution of bone material and its properties within the entire structure. Hence, QCT-based FEA can include the factors that influence the hip/femoral fracture. However, a large number of CT based 3D FE analyses have been conducted on proximal femur to predict bone fracture risk, where von Mises failure criterion based on distortion energy theory [19–23] has been adopted regardless of its limitation of predicting other than yielding in isotropic ductile materials. Therefore, the goal of this work is to set a criterion, which can lead us to justify the requirement of mono or bilateral hip analysis for assessing hip fracture more reliably using FEA. In this preliminary study, we only considered femoral neck region for assessing and comparing the fracture risk indicator (FRI). In this work, the mechanics of femoral neck fracture due to stress variations considering the maximum tensile and compressive stresses generated at the neck owing to the single-stance and sideways fall have been investigated on both left and right femora. The hip fracture risk has been assessed in terms of FRI, $\eta$, a ratio of bone stress to strength. Therefore, the comparison of FRIs of left and right femora will justify the requirement of bilateral fracture risk assessment.

The specific objectives of this paper are to: (1) conduct the patient-specific FEA of both left and right femora for the single-stance and sideways fall configurations; (2) compare the fracture risk indicators at the femoral neck of both the femora to investigate the justification of bilateral hip assessment using CT based FEA.

## Subjects and methods

### Subjects

In this study, QCT-scan images of 30 anonymous Canadian male and female have been selected randomly. The CT dataset of both left and right femora of the patients was obtained in Digital Imaging and Communications in Medicine (DICOM) format, removing all personal information as required under human research ethics approval, from the Great-West Life PET/CT Centre located at the Health Science Centre, Winnipeg, Canada. The mean age of the subjects was 63.7 ± 7.8 years, within the ranges of 51–78 years. Most of the patients (63%) were 60 and above, and the rest were below 60. The mean weight of the patients was 82.7 ± 16.8 kg, which varied between 51.7 to 111.4 kg.

### Imaging

The images were scanned by SIEMENS S5VB40B CT scan machine and acquired in the Great-West Life PET/CT at Winnipeg. The acquisition and reconstruction parameters were 120 kVp, 244 mAs, and image matrix of 512 × 512 pixels. Both high and low-resolution protocols with a slice thickness of 1 and 3 mm, respectively, were used with in plane spatial resolutions varying between approximately 0.78 and 0.98 mm. However, 1 mm slice thickness is more suitable for constructing a 3D model of a femur from the CT dataset by extracting the femur from pelvis, ensuring proper segmentation. The local bone density—expressed in Hounsfield Unit (HU)—correlated with CT voxel results in an inhomogeneous density distribution. A calcium hydroxyapatite calibration phantom (Mindways Inc., Austin, TX, USA) was incorporated during the time of image acquisition to correct the scanner drift and to attain an accurate estimation of bone mineral density.

### FE modelling of 3D reconstructed bone

Medical image processing software-Mimics® 16.0 (Materialise N.V., Leuven, Belgium)—has been used to generate the three-dimensional geometry of each femur from its DICOM image dataset. The 2D slices are stacked and converted into a 3D model using the interpolation algorithm embedded in Mimics. The 3D anatomy of a bone is extracted through careful segmentation, excluding soft tissue prior to the generation of a FE meshed model of the femur. The FE mesh is generated using the 3-matic module in Mimics (Fig. 3b). The 4-node linear tetrahedral element has been used in this study. The tetrahedral element can simulate irregular and complex geometric models such as those produced from various CAD/CAM systems. The element has four nodes with six degrees of freedom at each node: translations in the nodal x, y, and z directions and rotations about the nodal x, y, and z directions.

The FE mesh quality was checked and edited by using the mesh processing tools of Mimics. To investigate model convergence, FE models with different maximum element edge lengths were created. The generated mesh is then exported to MATALB® 14.0 (The MathWorks Inc, Natick, MA), where in-house built codes have been used to map material properties and to conduct the FEA. For each FE model, the maximum von Mises stress at the smallest femoral neck cross-section was calculated under the same loading and boundary conditions. The maximum element edge length that produced converged finite element solutions [24] was obtained and used in all the rest FE simulations. Hence, an adequate mesh density has been conserved to achieve model convergence.

### Material properties and its mapping on the FE model

Although bone is anisotropic [25, 26], the widely accepted existing empirical modulus-density relationships consider bone to be isotropic in literature [22, 27–30]. In this work, the properties of bone material have also been considered isotropic and inhomogeneous to commensurate with a reliable modeling approach. The material properties can be mathematically derived from CT data [31–35] by correlating HU and CT gray values. However, material models for bone either directly depend on the HU number [28, 36] or on the apparent density [31–34, 36]. In this work, density based material models have been considered to estimate Young’s modulus and compressive yield strength of a femur [31, 32], and they are,

1 $$E = 10,500\rho_{ash}^{2.29} \,\left( {\text{MPa}} \right)$$

2 $$\sigma_{{y_{C} }} = \left\{ {\begin{array}{*{20}c} {137\rho_{ash}^{1.88} \,\left( {\text{MPa}} \right),\quad \rho_{ash} < 0.317\,\left( {{\text{g/cm}}^{ 3} } \right)} \\ {114\rho_{ash}^{1.72} \,\left( {\text{MPa}} \right),\quad \rho_{ash} > 0.317\,\left( {{\text{g/cm}}^{ 3} } \right)} \\ \end{array} } \right.$$

where $\rho_{ash}$ is the bone ash density and is related to the equivalent $K_{2} HPO_{4}$ density $\left( {\rho_{{K_{2} HPO_{4} }} } \right)$ correlating *HU* by empirical equations [34, 35]. The tensile yield strength [37] is

3 $$\sigma_{{y_{T} }} = 0.8\sigma_{{y_{C} }}$$

A constant Poisson’s ratio of 0.4 is assumed for the bone material in the present analysis [34, 38]. A detail procedure to obtain the isotropic inhomogeneous material properties can be found in [39] and the references cited there. A brief description of the material mapping on to the FE mesh has been described below.

**Fig. 1:**
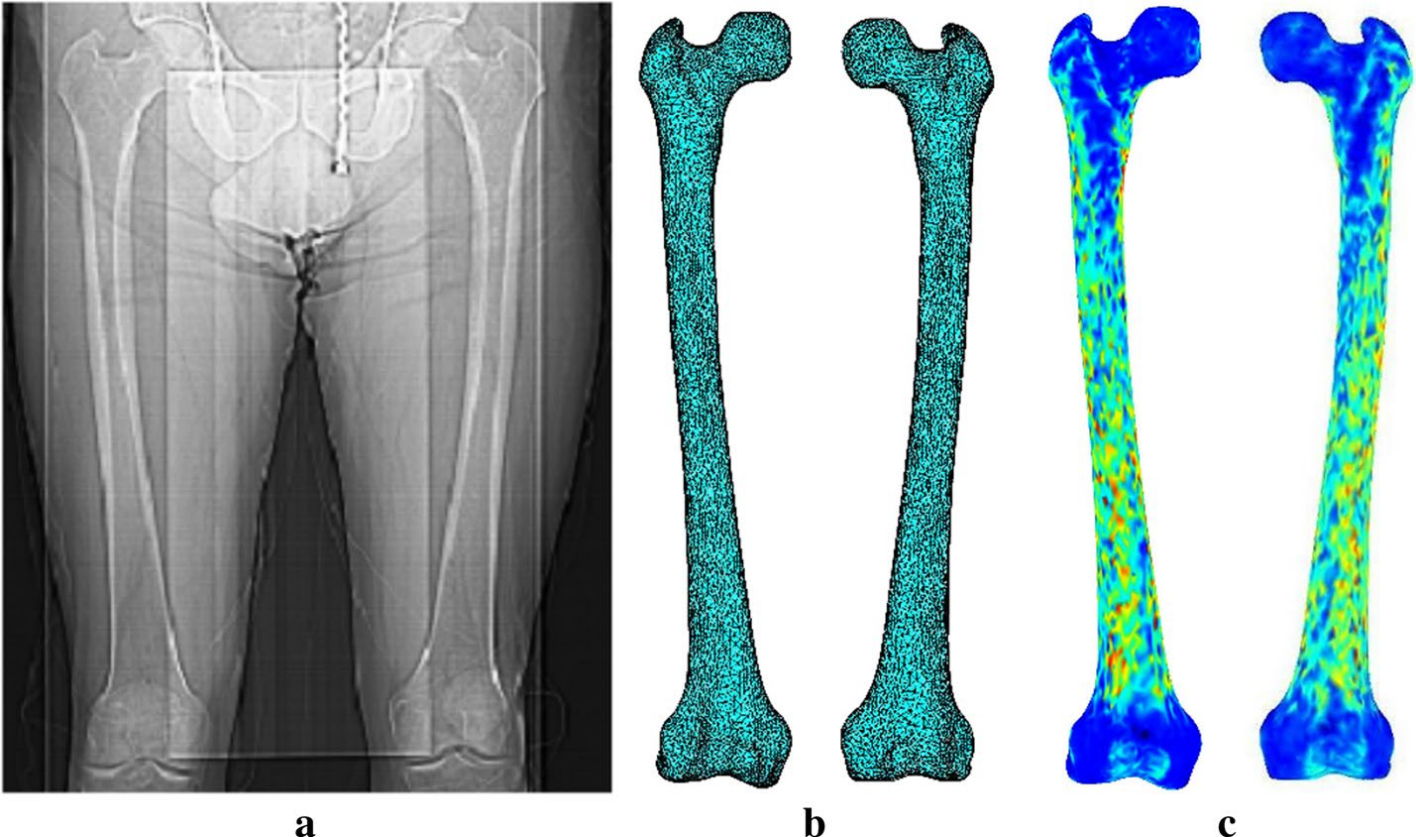
**a** A QCT image showing right and left femurs of a patient including pelvis and muscle, **b** 3D FE meshed models of right and left femora, **c** isotropic inhomogeneous material properties distributions in the femora

The material properties have been mapped following a node-wise approach [39] instead of element-wise [40–42] in the current work. This mapping approach is beneficial, because only one material definition is required depending on the assigned densities and resulting in a cost-effective and time-saving calculation. Nodal coordinates of the FE mesh are retrieved using the in-house built mapping algorithm. Each nodal coordinate is determined from the stack of CT slices considering the location of transverse plane (x and y coordinates of each pixel) and the axial height (z values), and the corresponding material data are mapped on to the nodes. To accommodate error due to the conversion of node coordinates, from the spatial coordinates (x, y, and z) into pixel indices (i and j), the HU at the corresponding locations in the CT slices are averaged within a definite zone surrounding the nodal coordinates. A longitudinal QCT image of both right and left femora of a patient, corresponding 3D meshed models, and the distribution of isotropic inhomogeneous material properties throughout the femoral bones are shown in Fig. 1. It is to be noted that the patient specific distribution of modulus might vary considerably depending on many factors such as age, sex, and even non-uniform bone loss between femurs of each patient.

### Loading and boundary conditions

**Fig. 2:**
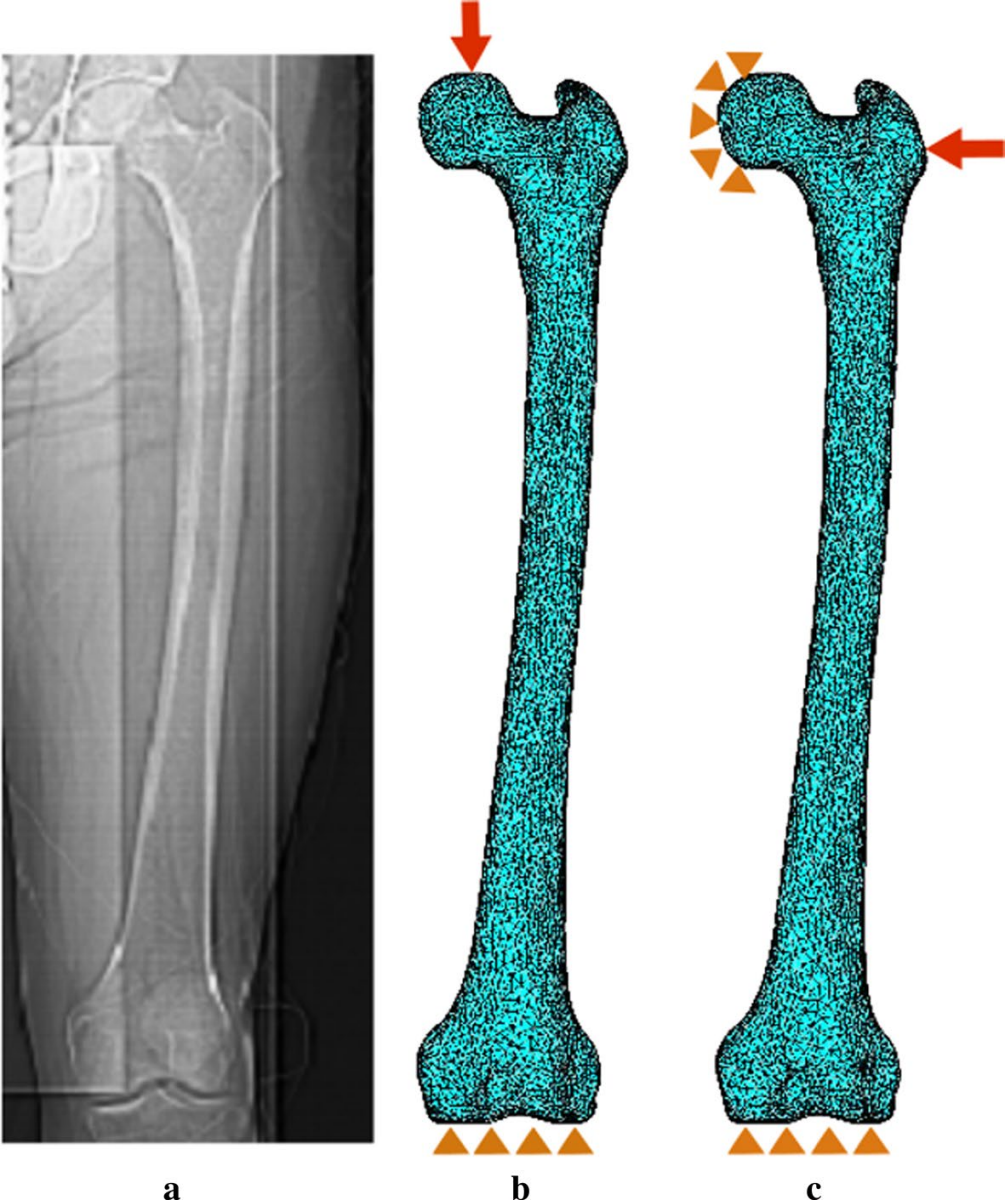
Boundary conditions of the QCT-based finite element modeling—**a** a QCT scan of a representative subject’s left femur, **b** single-stance load, **c** sideways fall load

Two loading conditions— single-stance and sideways fall, have been adopted to assess and compare the fracture risk of left with right hips. The fall typically results in the development of a peak stress at the femoral neck. A vertically downward load is applied to the superior surface of femoral head (Fig. 2b) for the single-stance configuration, whereas a lateral load at the greater trochanter, while constraining the nodes of the femoral head, simulates the sideways fall (Fig. 2c). The nodes at the distal condyles of the femur are completely constrained in all three directions for both the configurations [43–45]. Although the magnitude of load in one-legged stance is reasonably less than the sideways fall in real life [46, 47], in this work, an equal magnitude of load is applied to both the configurations to explore the effect of sideways fall, which frequently causes the fracture by developing stress variations in the femoral neck region.

## Results

Femoral neck is the most critical and vulnerable section, where tensile and compressive stress becomes higher for both the loading configurations and is, therefore, considered in the present work. The femoral neck undergoes constant bending during the one-legged stance, developing tensile and compressive stress at the superior and inferior surfaces of the femoral neck (Fig. 2b). From structural viewpoint, downward force through the femoral head causes tensile stress in the superior surface of the neck and compressive stresses inferiorly, which result in highest stresses in sub-capital and mid-femoral neck regions. On the contrary, the stress patterns are reversed in the neck region during the sideways fall due to lateral impact on the greater trochanter as shown in Fig. 2c. The fall consequently develops large compressive stress at the superior-posterior surface of the neck and tensile stress in the inferior region. Therefore, depending on the state of loading, the principal stresses, $\sigma_{1}$ and $\sigma_{3}$, are considered at the superior and inferior surfaces of the femoral neck region. The ratio of the maximum tensile and compressive stresses to appropriate tensile and compressive yield strengths given by Eqs. (2) and (3) have been considered to predict the fracture risk, and the associated fracture risk indicators are expressed as [39].

4a $$\eta_{T} = {{\left| {\sigma_{1} } \right|} \mathord{\left/ {\vphantom {{\left| {\sigma_{1} } \right|} {\sigma_{Y}^{T} }}} \right. \kern-0pt} {\sigma_{Y}^{T} }}$$

4b $$\eta_{C} = {{\left| {\sigma_{3} } \right|} \mathord{\left/ {\vphantom {{\left| {\sigma_{3} } \right|} {\sigma_{Y}^{C} }}} \right. \kern-0pt} {\sigma_{Y}^{C} }}$$

**Fig. 3:**
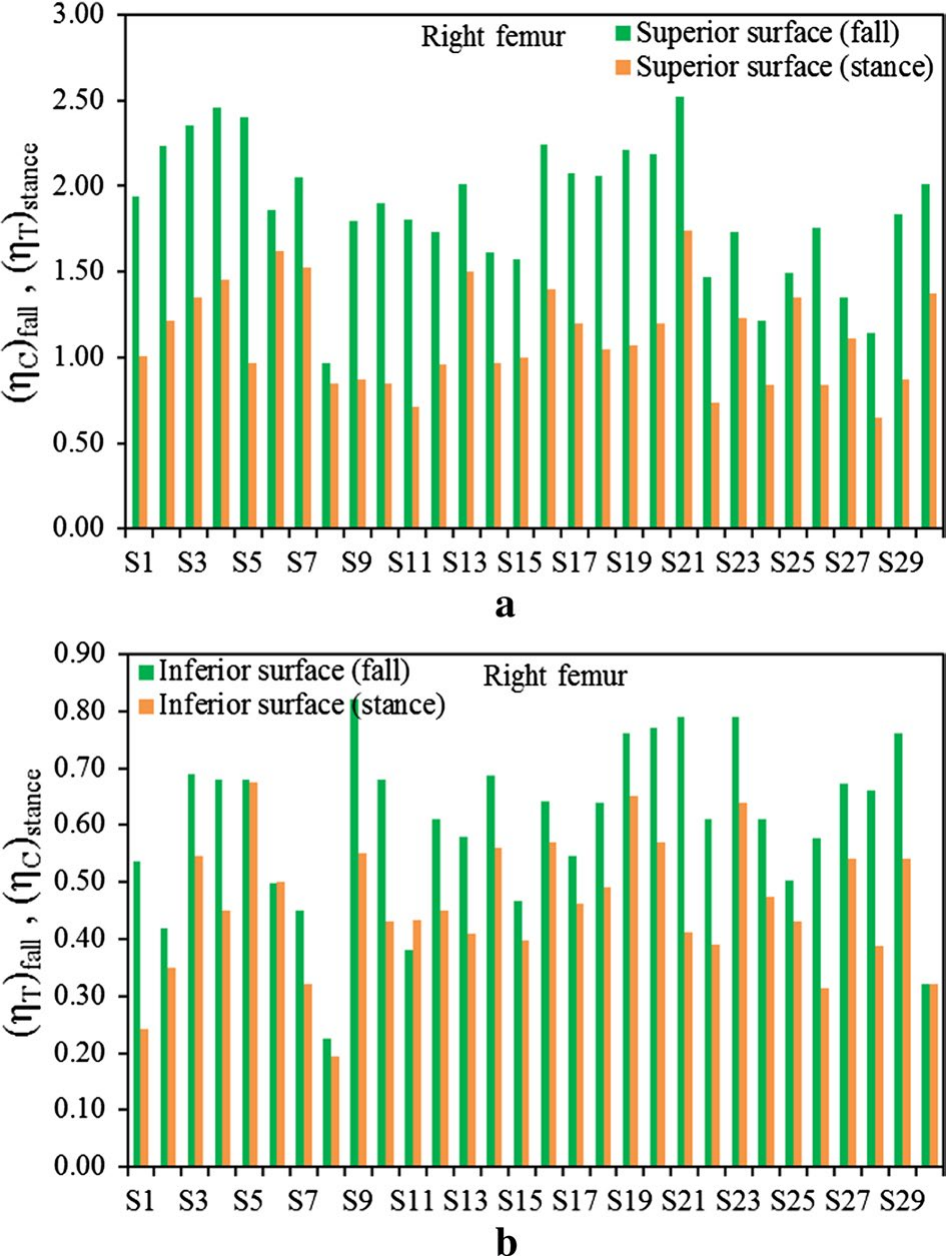
**a** Comparison of $\left( {\eta_{T} } \right)_{stance}$ and $\left( {\eta_{C} } \right)_{fall}$ at the superior surface, **b** the comparison of $\left( {\eta_{C} } \right)_{stance}$ and $\left( {\eta_{T} } \right)_{fall}$ at the inferior surface of femoral neck of right femur during single-stance and sideways fall configurations

**Fig. 4:**
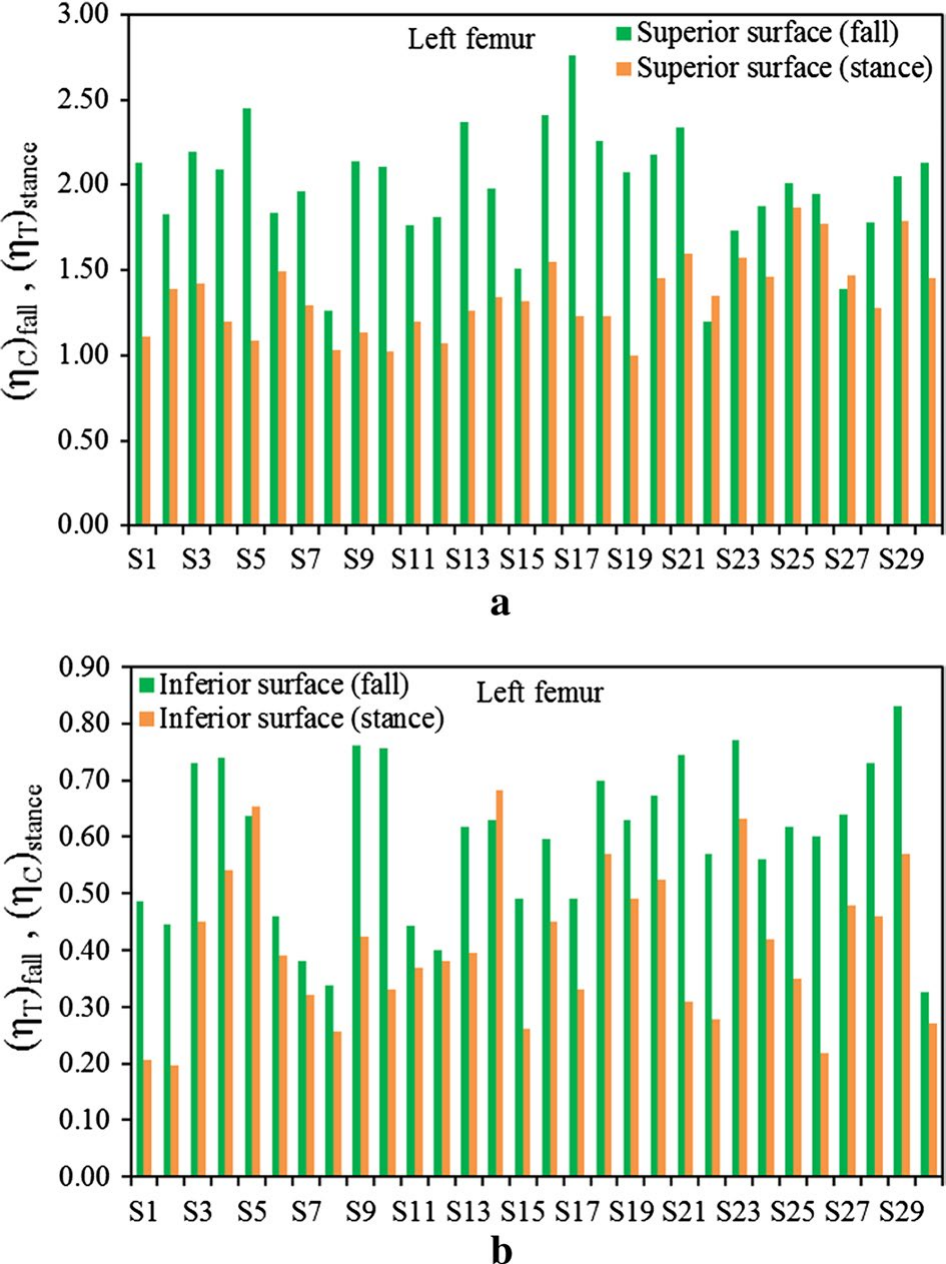
**a** Comparison of $\left( {\eta_{T} } \right)_{stance}$ and $\left( {\eta_{C} } \right)_{fall}$ at the superior surface, **b** the comparison of $\left( {\eta_{C} } \right)_{stance}$ and $\left( {\eta_{T} } \right)_{fall}$ at the inferior surface of femoral neck of left femur during single-stance and sideways fall configurations

**Fig. 5:**
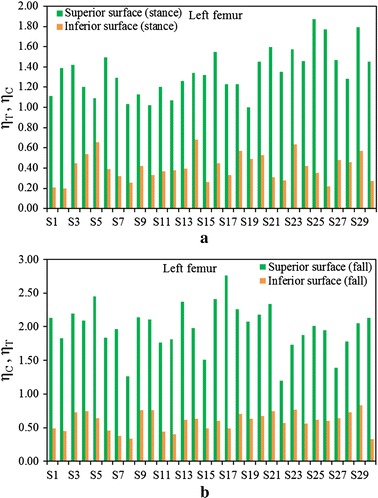
Comparison of $\eta_{T}$ and $\eta_{C}$ during single-stance (**a**) and the comparison of $\eta_{C}$ and $\eta_{T}$ during sideways fall (**b**) configurations at the superior and inferior surfaces of left femur

**Fig. 6:**
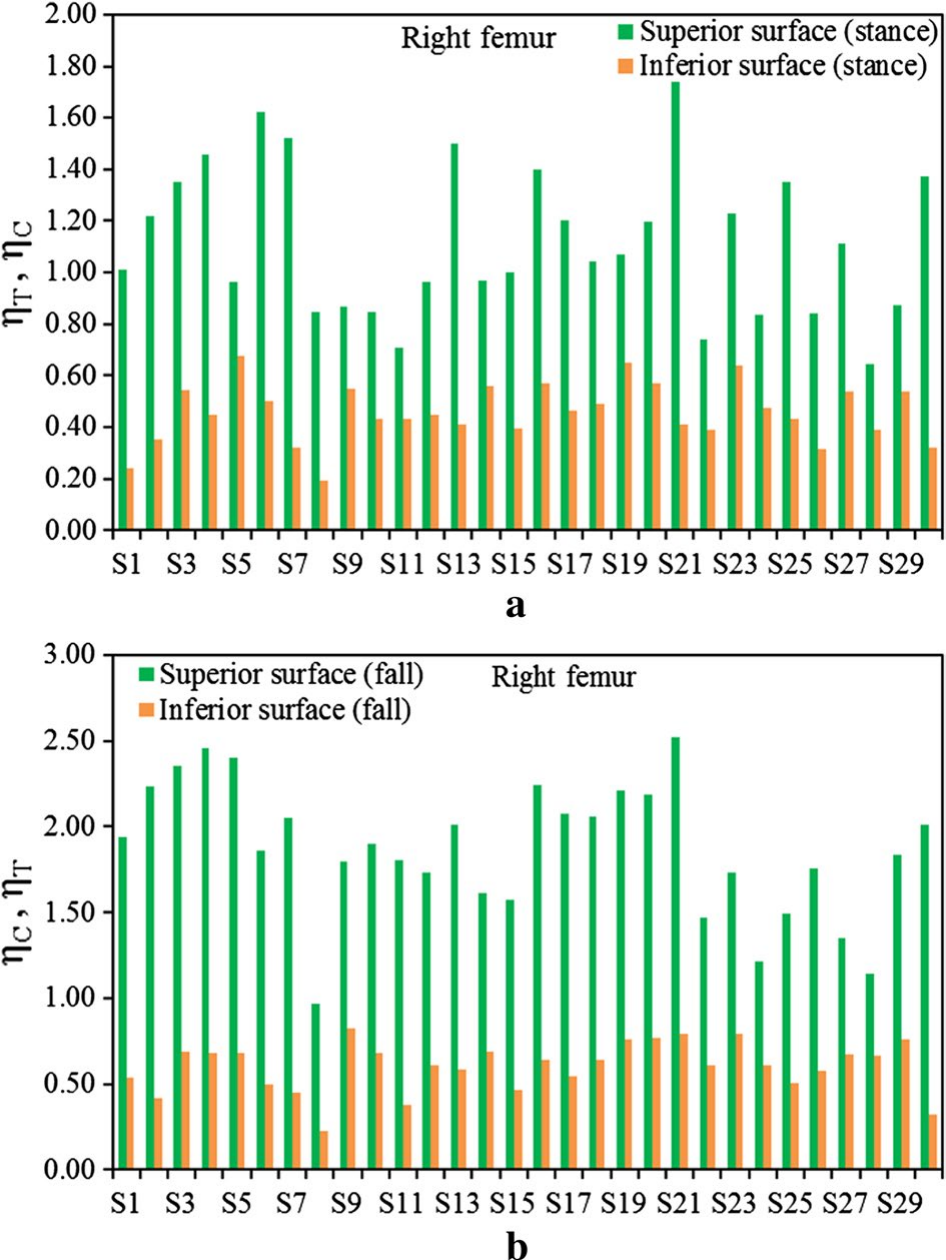
Comparison of $\eta_{T}$ and $\eta_{C}$ during single-stance (**a**) and the comparison of $\eta_{C}$ and $\eta_{T}$ during sideways fall (**b**) configurations at the superior and inferior surfaces of right femur

Figure 3a shows a comparison between $\left( {\eta_{T} } \right)_{stance}$ and $\left( {\eta_{C} } \right)_{fall}$ at the superior aspects of the femoral neck, and Fig. 3b displays the fracture risk indicators, $\left( {\eta_{C} } \right)_{stance}$ and $\left( {\eta_{T} } \right)_{fall}$, at the inferior aspects of the femoral neck of the right femur for the one-legged stance and sideways fall configurations, respectively. Figures  4a, b show the similar comparisons for the left femur. It is apparent that $\left( {\eta_{C} } \right)_{fall}$ is significantly higher than $\left( {\eta_{T} } \right)_{stance}$ for both left and right femora even the magnitude of applied load is same for both the loading configurations. Comparing the sideways fall and single-legged stance, the fracture risk indicators vary by 64 to 74% at the superior aspects and by 14 to 19% at the inferior surfaces of both femurs. Therefore, the results of this in vivo analysis clearly substantiate that the fracture is expected to initiate at the superior surface of femoral neck region if a patient experiences the impact of fall; and the fracture at this surface is as well observed both clinically and experimentally [48, 49]. The variations of FRI in the superior and inferior aspects of left and right femurs are shown in Figs. 5 and 6, respectively. The variation of FRI in the superior and inferior aspects of left and right femurs may reasonably manifest that the location of the impact load and its direction are largely responsible for hip fracture in different magnitude between the femurs and obviously more severe in actual scenario as the magnitude of fall load is much higher than the one-legged stance.

**Fig. 7:**
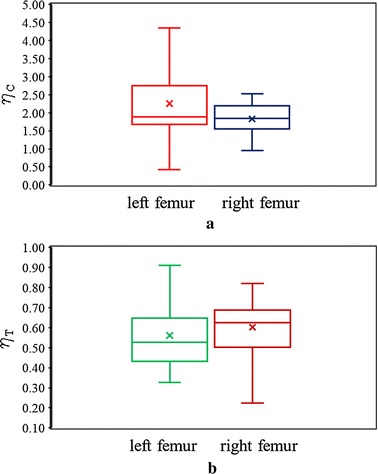
The distribution of FRI and its variability between left and right femurs during sideways fall at superior aspects (**a**) and inferior aspects (**b**)

**Fig. 8:**
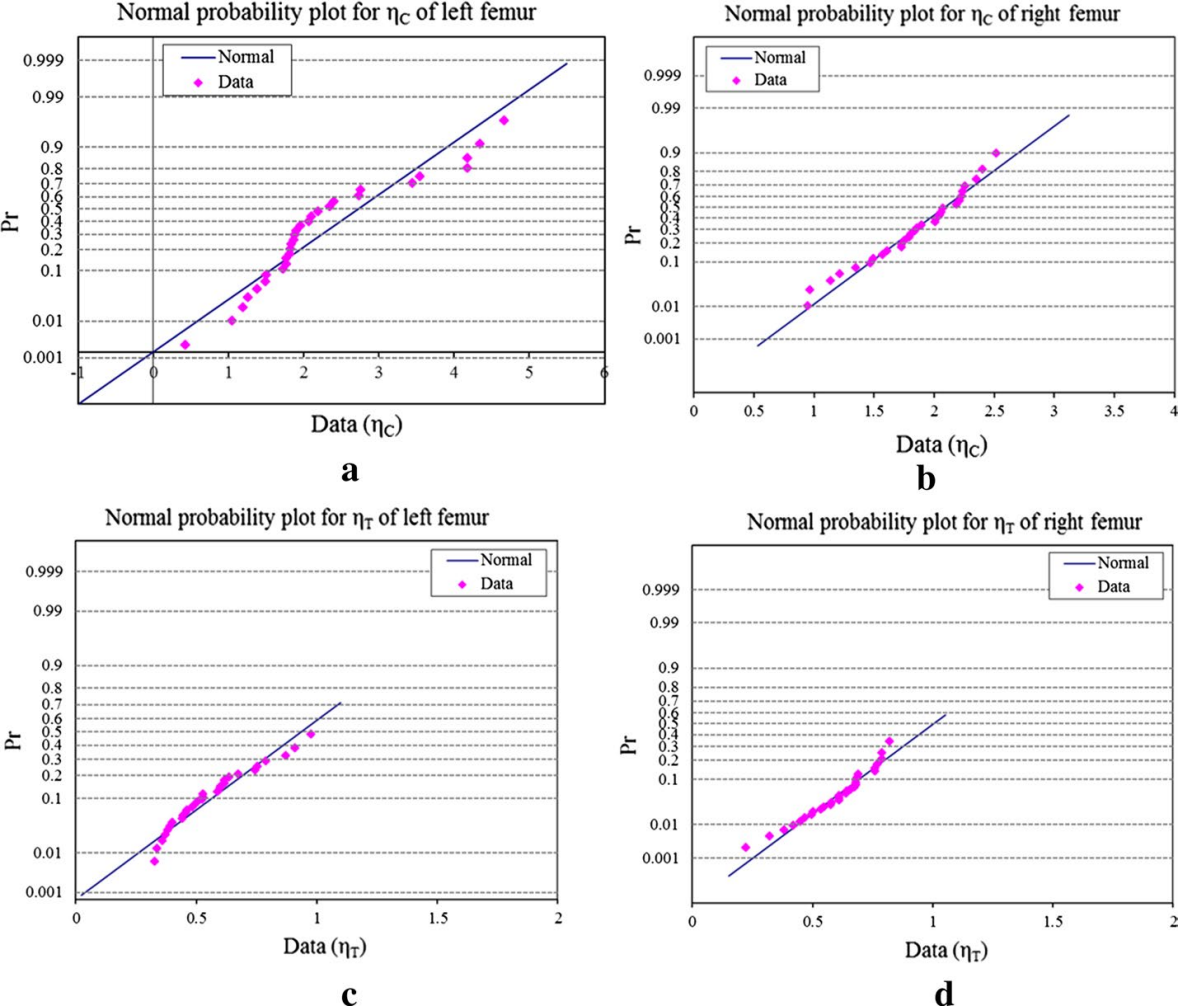
Comparison of distributions of $\eta_{C}$ at the superior surface of left (**a**) and right (**b**) femurs, and distribution of $\eta_{T}$ at the inferior surface of left (**c**) and right (**d**) femurs using Anderson–Darling normality test

The statistical distribution of FRI and its variability during fall at the superior and inferior aspects of left and right femurs have also been shown in Fig. 7. Figures 7a and b show box and whisker plots of FRI at the superior and inferior aspects of the femoral neck of left and right femurs, respectively. The upper and lower boundaries of the box indicate upper (75th percentile) and lower (25th percentile) quartile, whereas the internal line indicates the median FRI; and the cross sign represents the mean FRI for each dataset. The lines extending vertically from the boxes, known as whiskers, illustrate the variability outside the upper and lower quartiles. The box and whisker plots in Fig. 7a describe that the distribution of FRI at the superior aspect of left femur is left-skewed, whereas the FRI of the right femur follows a near normal distribution. The distributions of FRI at the superior surface of the two femurs are further tested and compared using Anderson–Darling normality test in Fig. 8, which shows that the FRI distribution of left femurs in Fig. 8a rejects normality (*p* ≤ 0.05), whereas the distribution in Fig. 8b exhibits normality for the right femurs. Figure 7b also resembles near normal distributions of FRIs at the inferior aspects of left and right femurs, respectively. The normality of the FRI distribution at the inferior surface of left and right femurs is also supported by the Anderson–Darling test shown in Fig. 8c and d, respectively. It is evident that the probability of fracture and its variability is significantly different between the two sides.

## Discussion

The non-invasive procedure of developing a CT based FE model, considering 3D bone geometry, material properties, and loading effect can be an excellent tool for estimating a priori the in vivo hip fracture risk of geriatric population and osteoporotic patients. The maximum and minimum principal stresses have been found to be the characteristic of sideways fall in comparison with one-legged stance. Total body weight is supported by a femur during the one-legged posture, whereas the sideways fall from a standing height impacted on a higher load than the body weight. Hence, the simulations with different amount of loads may be mimicking the two configurations but lack a conclusive correlation between the resultant stress distributions [46, 47, 50, 51] for predicting the fracture risk. Therefore, an equal magnitude of load is considered for both the configurations to distinguish the effect of sideways fall from the stance in conjunction with bone quality and thereby evaluating the fracture risk and its variation between the femurs, if there is any.

The physiology of bone demonstrates that bone typically comprises different tensile and compressive strength due to which bone loss preferentially occurs at the superior aspect of the femoral neck [47]. The inferior cortex of the femoral neck is usually thicker than the superior, and the thinner superior cortex could make the femoral neck susceptible to failure, while being subjected to greater compressive stress in consequence of the impact of fall. It is obvious that the effect of stress is higher at the weaker region—the region with lower mass density—and is demonstrated in Table 1 for both types of femur.

**Table 1:** Average variation of FRIs $\left( {\eta_{T} ,\eta_{C} } \right)$ at the superior and inferior aspects of left and right femora during single-leg stance and sideways fall configurations

Femur type	Single-legged stance		Sideways fall	
	Superior surface	Inferior surface	Superior surface	Inferior surface
Left femur	1.83 ± 0.56	0.41 ± 0.13	2.26 ± 0.56	0.50 ± 0.21
Right femur	1.57 ± 0.29	0.46 ± 0.12	1.78 ± 0.42	0.60 ± 0.15

**Fig. 9:**
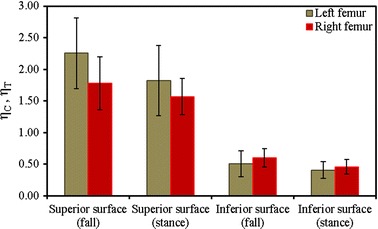
Comparison of fracture risk indicators between left and right femur during single-stance and sideways fall configurations at the superior and inferior surfaces of femoral neck region

For both left and right femurs, the fracture risk indicators, $\eta_{T}$ and $\eta_{C}$ are considerably higher at the superior cortex, where more bone loss is generally observed, than the inferior one. The average variation of FRI during single-leg stance and sideways fall configurations for left and right femora has been shown in Fig. 9. For the single-leg stance, the average variations of FRI between the left and right femurs are 11% at the inferior surface and 16% at the superior surface, whereas the FRIs vary between 17 and 27% for the same during the sideways fall. However, the variation of FRI between the femurs of an individual patient can be considerably high and may not be truly reflected by mean variation. In our study, the maximum individual variation of FRI between the left and right femurs has been found up to 91% at the superior surface and 50% at the inferior surface. Since the lesser bone loss makes the inferior cortex thicker, the variation of FRI is less in this location. On the contrary, the thinner superior cortex is perceptibly responsible for higher variation of FRIs at the superior surfaces of the neck. The variation between the left and right femurs may be originated from the non-uniform osteopenia or osteoporosis. Obviously, both femora may not experience similar degree of bone loss or not suffer osteoporosis in a similar manner. Moreover, any structural differences in femur length, shape and size may also be responsible for such variation between the femurs.

**Fig. 10:**
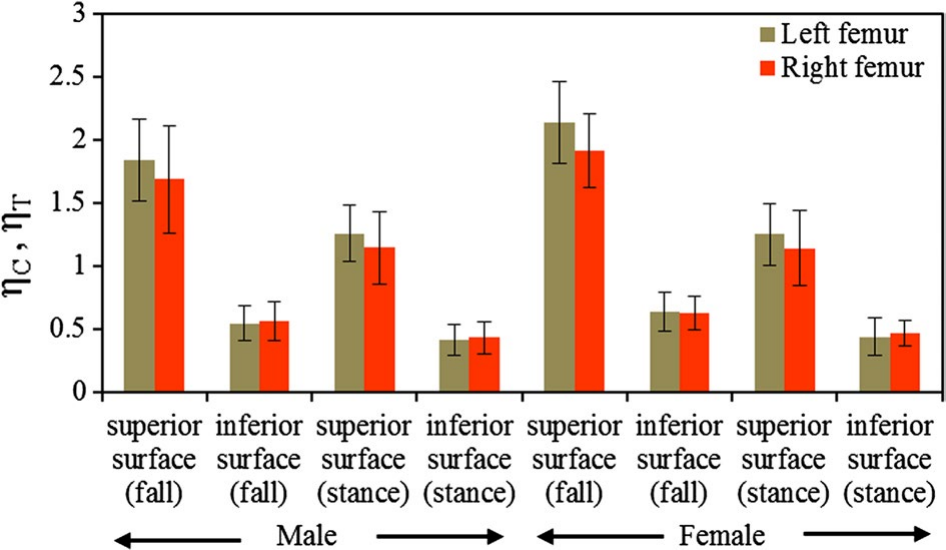
Comparison of FRI at the superior and inferior surfaces of femoral neck of the left and right femora of male and female during single-stance and sideways fall configurations

The FRIs between the left and right femur of men and women have also been compared in Fig. 10. The FRIs at the superior aspects of the left and right femora of male vary by approximately 15 and 11% during fall and one-legged stance, respectively. For female subjects, these variations are approximately 23 and 11% for the respective loading conditions. Since the inferior cortex is less likely to suffer bone loss, the mean FRIs are nearly equivalent and varied by less than 4% in this region. It is clearly evident that the degeneration of bone between the femurs are not uniform that causes variations in FRIs between the hips. Hence, the clinical practice of densiometric screening of only one leg, considering the good correlation of BMD between the left and right femurs are judgmental. The difference in FRI between male and female subjects during fall not only helps us speculating the severity of the osteoporotic hip fracture in women health, but the variation also corroborates the fact that women are more likely to suffer osteoporotic hip fracture than men owing to age and physiological factors such as menopause. The hormonal imbalance and loss of calcium may also affect the BMD loss in a nonuniform manner more severely to women than men and consequently the hip fracture risk.

In addition to the distribution of FRI at left and right femurs (Figs. 7, 8), the strength of association between the FRIs of the left and right femora due to fall has been statistically compared using *paired sample t*-*Test*. This test typically provides a measure of the separation of data in any two sets and a measure of confidence if those data reflect real (significant) differences or not. The *p* value of two sample *paired t*-*Test* of FRI at the superior aspects of left (N = 30) and right (N = 30) femurs is 0.02 and *p* ≤ 0.05; and the *t*-value is 2.43. Therefore, there is a statistically significant difference between FRIs of left and right femora at the superior aspects, left femur (mean = 2.26, SD = 1.03) and right femur (mean = 1.83, SD = 0.41), p ≤ 0.05. Therefore, we reject the null hypothesis that there is no difference in FRI distribution between left and right femurs. On the contrary, for the inferior aspects of the left and right femurs, we have found *t* value is 1.43 and *p* value is 0.16, i.e. *p* ≥ 0.05. Therefore, we fail to reject the null hypothesis that there is no difference in FRI at the inferior aspects between left and right femurs. As noted earlier, the superior aspect is the most vulnerable location, where the fracture typically initiated due to fall, and we are more concerned on assessing hip fracture at this location. A larger population based study with specific age and sex group can give a better insight into the justification of bilateral hip scanning.

The tension and compression of superior and inferior surfaces are also affected by other factors such as the orientation of trabecular and femur, diameter and length of the femoral neck, the angle of impact, and impact site, which also require further investigation to get an insight into the difference of FRI and its cause in left and right femora, if any. Nevertheless, the current work on assessing hip fracture risk has been conducted on continuum level, which is unable to give insight into the effect of bone microstructure that leads to bone yielding, damage, and fracture at the organ level [52]. Due to aging, disease process and the influence of therapeutic agents, bone loss occurs. A more rod-like structure of trabecular bone is, therefore, observed in the femoral neck, which thereby is likely to be more susceptible to bending and buckling failure modes. Connectivity density (trabecular connectivity) also decreases and influence the hip fracture as well [53]. With ages, cortical pores fuse together to form giant pores [54, 55], which could be considered a pivotal loss of cortical thickness and strength and leads to fracture triggered by fall. However, the effect of bone microstructure in organ level fracture can be captured by multiscale modeling, which is beyond the scope of current work. Moreover, it is not known if the subjects of the dataset considered in this study are healthy or diagnosed with osteoporosis based on existing assessment criteria. A similar study with known dataset of osteoporotic patients can provide us a more conclusive result of the variability of the FRIs between the left and right hips and will be conducted in future.

The goal of the current work is to study the difference in FRI between the left and right femur by investigating the effect of fall, comparing both single-stance and sideways fall configurations, to justify the need for bilateral hip scanning for predicting hip fracture risk. Comparing to BMD measurement time of 20 min in early 1980s with dual-photon absorptiometry (DPA), today’s imaging modalities take few minutes for a single hip scanning in automated sequential bilateral hip mode and adds a minute or so for the second hip. Hwang et al. found a considerable difference in femoral neck BMD between the right and left sides [17], which would be inaccurate if the measurement was done unilaterally. The left–right discordance has been found up to 14% at the femoral neck of 28% of 537 subjects examined by Cole and Larson [56]. The left–right difference in BMD exceeded the Smallest Detectable Difference (SDD) of 0.024 g/cm^2^ for 52.1% participants of a total of 3481 patients at femoral neck [57]. The variation of left–right FRI can be originated from lifestyle, physical activities, alcohol consumption and can be influenced by the use of medication. Hence, the clinical significance of bilateral assessment of predicting hip fracture is obvious. In general, the radiation dose (even this is low now a days) as well as the cost restrict the bilateral hip scanning done clinically, but the cost would be minimal as repositioning is often not required for imaging the second femur. Moreover, the computational analysis will be an effective tool for assessing bilateral hip fracture risk in terms of cost and time and will lead to a more efficient and clinically accurate assessment. This preliminary study, however, shows the variability of FRIs between the left and right femurs and justifies that bilateral hip scanning in predicting hip fracture is necessary for more accurate prediction of hip fracture risk.

## Conclusions

The QCT-based finite element modeling demonstrates an important route to assess hip fracture to elderly people, who specially suffer from osteoporosis. *A priory* assessment of hip fracture is a prerequisite to prevent the fracture to not only lower the treatment cost but also to lessen the sufferings. The patient specific maximum principal stress based fracture risk indices, $\eta_{C}$ and $\eta_{T}$, have been evaluated for both left and right femurs to investigate the correlation and/or difference between the FRIs of the femurs. The variability of FRI distribution and lower *p* value of two *paired sample t*-*Test* indicate strong difference between the FRI of the two femurs during fall. However, the variation of FRI of an individual patient could be even significantly large. Therefore, a bilateral hip scanning is required to prevent, detect and to treat the hip fracture risk more accurately.
